# The Effects of Salt and Glucose Intake on Angiotensin II and Aldosterone in Obese and Nonobese Patients with Essential Hypertension

**DOI:** 10.1155/2020/6017105

**Published:** 2020-03-19

**Authors:** Nouralsalhin Alaagib, Mohammed Sukkar, Mohammed Kardash

**Affiliations:** ^1^Department of Physiology, Faculty of Medicine, University of Khartoum, Khartoum, Sudan; ^2^Faculty of Medicine, Nile University, Khartoum, Sudan; ^3^Faculty of Medicine, Omdurman Ahlia University, Omdurman, Sudan

## Abstract

**Background:**

The exact mechanisms for the development of essential hypertension are not known. Activation of the renin-angiotensin-aldosterone system (RAAS) in adipose tissue may represent an important link between obesity and hypertension. This study investigates the effects of oral intake of glucose with and without NaCl on angiotensin II (AngII) and aldosterone in obese and nonobese patients with essential hypertension.

**Methods:**

Twenty newly diagnosed untreated essential hypertensive patients and 15 normotensive control subjects matched for age, gender, and BMI were studied. Participants fasted overnight (8–10 hrs), and then each subject took 75 gm glucose alone and with 3 gm NaCl, each dissolved in 250 ml. Subjects were monitored for 2 hours. Half hourly BP, plasma glucose (PG), serum Na^+^, K^+^, insulin, AngII, and aldosterone were measured. Subjects were classified into obese (BMI >30 Kg/m^2^) (11 patients and 8 control) and nonobese (BMI <30 Kg/m^2^) (9 patients and 7 control).

**Results:**

After intake of glucose with NaCl serum, AngII was significantly higher in obese hypertensive patients compared with nonobese patients (*P* = 0.016). Intake of glucose with NaCl resulted in a significantly higher serum Na in obese hypertensive patients compared with nonobese patients Na (*P* = 0.009). Serum aldosterone was significantly higher in obese patients (*P* = 0.03, after glucose; *P* = 0.003, after glucose with NaCl) and in nonobese patients (*P* = 0.000 and *P* = 0.000, respectively) compared with their respective normotensive control subjects. In obese and nonobese patients, intake of glucose and glucose with NaCl showed no significant change in the levels of serum AngII and aldosterone which was associated a significant increase in serum Na in obese patients (*P* = 0.03) and a highly significant reduction in serum K in nonobese patients (*P* = 0.001).

**Conclusion:**

Failure of suppression or inappropriate maintenance of secretion of AngII and aldosterone in both hypertensive groups by intake of glucose with NaCl may indicate a possible mechanism of essential hypertension.

## 1. Introduction

More than 25% of the world adult population has hypertension [1]. The exact mechanisms for the development of essential hypertension are not known. A number of factors have been described to be associated with hypertension such as increased dietary sodium [2], hyperinsulinemia [3, 4], obesity [5], and hyperglycemia [6]. Obesity-related hypertension is commonly associated with other elements of the metabolic syndrome which increase the risk of life-threatening conditions such as kidney damage and heart failure [7]. It was suggested that activation of the renin-angiotensin-aldosterone system (RAAS) in adipose tissue may represent an important link between obesity and hypertension [8].

Angiotensin II (AngII), acting through the AT_1_ receptors, increases the generation of reactive oxygen species (ROS) in the vasculature, which may contribute to endothelial dysfunction and hypertension [9]. Huang et al. demonstrated that both circulating AngII and aldosterone act within the central nervous system to cause sympathoexcitation and raise the BP [10]. In addition to the effectiveness of AngII receptor blockers (ARBs) in treatment of hypertension, they have been found to reduce oxidative stress and inflammation [11], progression of arterial stiffness [12], and cardiac hypertrophy and improve renal function [13]. In addition to the well-known effects of aldosterone on renal handling of sodium and potassium and extracellular fluid volume expansion, aldosterone has rapid actions that are produced when it binds to mineralocorticoid receptors on the surface of the cells. These “nongenomic” actions of aldosterone most likely participate in the control of vascular resistance and may have a role in human hypertension and cardiovascular disease [14]. Old studies suggested that aldosterone may have a role in essential hypertension [15, 16].

We hypothesized that dietary intake of Na^+^ with glucose in patients with essential hypertension, at least in some patients, may be associated with changes in aldosterone and AngII, which results in high BP. This study was done to investigate the effect of oral glucose with or without NaCl on serum aldosterone and AngII in adult patients with essential hypertension.

## 2. Methods

This is a short-term experimental study which investigates the acute short-time effects of a single intake of glucose with and without NaCl, each on a separate day, on BP and RAAS. Long-term effects have not been investigated in this study. Twenty newly diagnosed untreated essential hypertensive patients and 15 normotensive control subjects matched for age, gender, and BMI were included in the study. Sample size was calculated using the formula for experimental study with serial samples (Equation 2 (Snedecor and Cochran1989) [17]):

(1) $$\begin{array}{c} n=1+2C\left(\frac{s}{d}\right)2. \end{array}$$

where *s* is the standard deviation, *d* is the difference to be detected, and *C* is a constant dependent on the value of *α* and *β* selected.

Hypertension was defined as having BP ≥ 140/90 [18]. Subjects were recruited from primary health care centers. Smokers and those with diabetes, cardiac or renal disease, or taking medications were excluded. Hypertensive patients with mandatory reasons for immediate initiation of treatment, e.g., very high BP, target organ damage, and hyperlipidemia were also excluded. After screening visits to identify and select new cases of essential hypertension, each subject underwent complete physical examination, and ECG was done for all participants. Each subject filled a questionnaire containing personal data and medical history and signed an informed consent form. This study was approved by the Ethical Committee of the Faculty of Medicine University of Khartoum. The study protocol conforms to the ethical guidelines of the 1975 Declaration of Helsinki as reflected in a prior approval by the institution's human research committee.

Weight (kg) and height (m) were measured for all participants, and BMI (kg/m^2^) was calculated as a ratio between body weight (kg) and squared height (m^2^). A venous blood sample was taken, and random blood glucose, urea, creatinine, and lipid profile were done for each subject to exclude any abnormality.

Subjects were advised to take their normal diets and not to restrict carbohydrates or salt intake. On the day of the experiment, subjects fasted overnight (8–10 hrs) and attended to the laboratory early morning. After resting for 15 minutes, baseline fasting BP and blood samples were taken.

All BP measurements were taken in sitting position using mercury sphygmomanometer (Kawamoto, Japan) by the investigator herself , according to the standardized methodology [18]. An appropriate-sized cuff was used, and a larger cuff was used for obese patients. SBP was taken as the point of onset of the auscultated pulsation (phase 1), and diastolic BP (DBP) was the point just before the disappearance of the sounds (phase 5). Three readings were taken at intervals of at least 1 minute, and the average of those readings was used for statistical analysis. If there is >5 mmHg difference between the first and second readings, an additional reading was obtained, and then the average of these multiple readings was used [19].

Each subject underwent a set of 2 experiments and took (i) 75 gm glucose solution and (ii) 75 gm glucose plus 3 gm NaCl dissolved in 250 ml of water to be consumed in no more than 5 minutes. The 3 gm NaCl is the maximum level of daily nutrient intake that is likely to have no risk of adverse effects [20]. Each experiment was scheduled on a different day separated by at least 3 days with random sequence of experiments for all participants.

Subjects were monitored in a sitting position in quite comfortable room temperature throughout the two-hour period of experiment. Half hourly BP measurements and venous blood samples were taken to measure plasma glucose using the glucose oxidase method (Biosystem, Spain) by using a spectrophotometer. The following blood chemistry tests were done: serum Na and K were measured by a Na/K analyzer (Easy Lyte Na/K analyzer, Medica, USA) using ion-selective electrode (ISE) technology.

Serum insulin was measured using insulin quantitative immunoassay test kits (Immunospec, USA) by Sandwiched ELISA, aldosterone was measured using enzyme immunoassay test kits (Immunospec, USA) by competitive ELISA, and AngII was measured by Sandwiched ELISA using Human Angiotensin II ELISA kits (WKEA MED SUPLPLIES CORP, USA). AngII was measured for hypertensive patients only.

### 2.1. Statistical Analysis

Results were saved and analyzed using the Statistical Package Program for Social Sciences (SPSS) version 22. Descriptive statistics was displayed as means ± standard error. Comparisons of the means between obese and nonobese patients and between patients and their control were done using the independent *t*-test. *P* value <0.05 was considered significant. To investigate the change in BP, plasma glucose, serum Na, K, AngII, and aldosterone after intake of glucose or glucose with NaCl for each measurement, the means of the five half hourly samples were compared using the ANOVA test; and results were considered significant when *P* value <0.05. To verify associations between BP, plasma glucose, serum Na, K, insulin, AngII, and aldosterone, Spearman correlation was done. The correlations were based on the means of the five samples taken half hourly through the period of the two-hour experiments.

## 3. Results

This study included 20 patients with essential hypertension; 11 were obese (BMI ≥30 Kg/m^2^) and 9 were nonobese (BMI <30 Kg/m^2^) [21]. The normotensive control group included 15 subjects matched for age, gender, and BMI; 8 were obese and 7 were nonobese subjects.

In the baseline fasting state, there was no significant difference in serum AngII or aldosterone between obese and nonobese hypertensive patients. However, serum aldosterone was significantly higher in nonobese hypertensive patients compared with nonobese control subjects (*P*=0.008) (Table 1) and in obese normotensive subjects than nonobese normotensive subjects (*P*=0.02).

### 3.1. Effect of Intake of Glucose and Glucose with NaCl on Serum AngII and Serum Na

AngII was measured only in hypertensive patients because AngII levels in most of hypertensive patients were at the lower limit of detection by the ELISA kit which was used to estimate AngII level. Therefore, we expected that AngII will be less in normotensive subjects, will decrease more after NaCl intake, and may not be detectable by the ELISA kits used.

**Figure 1:**
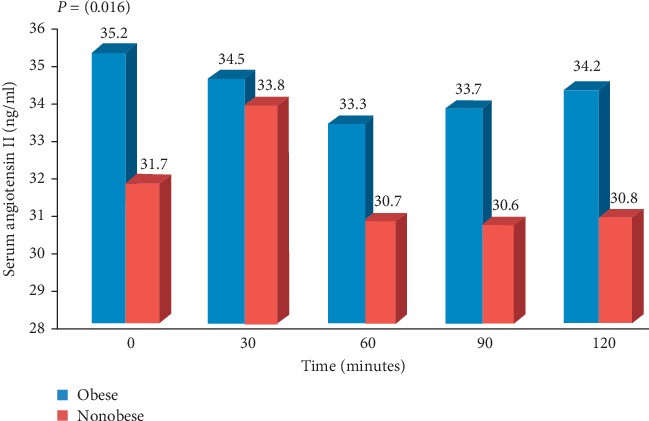
Serum angiotensin II in obese and nonobese hypertensive patients after intake of glucose with NaCl.

In both obese and nonobese hypertensive groups, serum AngII levels did not change significantly after intake of glucose or glucose with NaCl, in spite of significant increase in serum Na in obese patients (*P*=0.03) (Table 2). After intake of glucose with NaCl, serum AngII was significantly higher in obese hypertensive patients compared with nonobese patients (*P*=0.016) (Figure 1). The means of serum AngII for obese patients and nonobese patients are shown in Tables 2 and 3, respectively.

In obese hypertensive patients, intake of glucose with NaCl was associated with a significant increase in serum Na at 30 and 60 minutes (*P*=0.03) (Table 2). In addition, a significantly higher serum Na (*P*=0.009) was found in obese hypertensive patients (mean = 139.5 ± 0.4) compared with nonobese patients (mean = 137.5 ± 0.5) (Table 4).

In nonobese hypertensive patients after intake of glucose with NaCl, serum Na showed a significant positive association with plasma glucose (*P*=0.005) and serum insulin (*P*=0.006).

### 3.2. Effect of Intake of Glucose and Glucose with NaCl on Serum Aldosterone and Serum K

**Figure 2:**
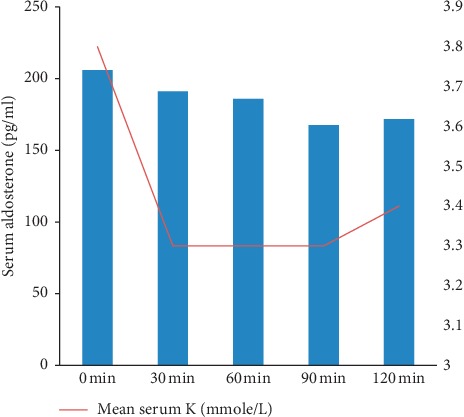
Serum aldosterone and serum K in nonobese hypertensive patients after intake of glucose with NaCl.

After intake of glucose with NaCl, serum aldosterone in obese and nonobese hypertensive patients did not change significantly (Tables 2 and 3) which was associated with a highly significant reduction in serum K in nonobese patients (*P*=0.001) (Figure 2).

In normotensive subjects, intake of glucose with NaCl resulted in a decrease in serum aldosterone which was significant in obese normotensive subjects (*P*=0.04).

Comparisons of the means of serum aldosterone between hypertensive patients and their matched controls after intake of glucose and glucose with NaCl showed that serum aldosterone was significantly higher in hypertensive patients compared with control subjects (*P* < 0.05).

In normotensive subjects, serum aldosterone levels were significantly higher in obese normotensive subjects than nonobese subjects after intake of glucose and glucose with NaCl (*P*=0.000) (Tables 4 and 5). However, in hypertensive patients, aldosterone levels were higher in nonobese than obese patients which were significant after intake of glucose alone (*P*=0.013) (Table 5).

After intake of glucose, aldosterone showed significant positive correlation with BP in obese hypertensive patients and nonobese normotensive subjects (*P*=0.000).

### 3.3. Comparison of the Effects of Intake of Glucose Alone and with NaCl

When we compared the effects of intake of glucose alone and with NaCl in obese hypertensive patients, we found no significant difference in BP, serum aldosterone, and AngII between intake of glucose and glucose with NaCl. However, in nonobese hypertensive patients, mean serum aldosterone was significantly higher (*P*=0.001) after intake of glucose (mean = 245.90 ± 16.15) than glucose with NaCl (mean = 184.46 ± 7.59). AngII levels were significantly higher after intake of glucose with NaCl (*P*=0.01) (mean = 31.56 ± 0.65) than after intake of glucose alone (mean = 28.10 ± 1.21).

## 4. Discussion

In this study after intake of glucose with NaCl, the levels of serum AngII were significantly higher in obese hypertensive patients compared with nonobese patients. Comparable results were reported by Engeli et al. who found that both levels of angiotensinogen and AngII were increased in visceral obesity [22]. Experimentally, a mouse model with transgenic overexpression of angiotensinogen in adipose tissue showed visceral obesity and hypertension [23]. It has been found that angiotensinogen, ACE, and AT_1_ receptor gene are widely expressed in human adipose tissue [24], and production of AngII and angiotensinogen in adipose tissue is suggested to be increased in obese subjects [25].

It is well known that AngII can increase sympathetic nervous system activity in humans and that the RAAS and sympathetic nervous system are linked by a positive feedback relationship which may induce hypertension in obese subjects. Moreover, the increase in sympathetic nervous system activity and the dysregulation of RAAS can occur when their inhibition by the cardiac natriuretic peptide system is decreased. Atrial (ANP) and ventricular (BNP) cardiac natriuretic peptides directly inhibit renin and aldosterone secretion, as well as sympathetic nervous system activity and vasopressin secretion [26]. ANP and BNP are cardiac hormones that work to reduce the BP. These hormones are able to induce natriuresis and diuresis, cause vasodilation, and antagonize the RAAS at multiple levels [26]. On the heart, they have antihypertrophy and antifibrosis activities [27, 28]. The cardiac natriuretic peptide system directly reduces renin and aldosterone secretion and antagonizes AngII and aldosterone effects on target cells [26]. A number of studies reported reduced levels of circulating natriuretic peptides in obese subjects with hypertension [29, 30] and without hypertension [31–33]. We found that in obese hypertensive patients, the intake of glucose with NaCl was associated with a significant increase in serum Na and that obese hypertensive patients had higher serum Na and AngII compared with nonobese patients. These findings support the hypothesis made by Engeli and Sharma (2000) that disturbed sodium handling appears to play a central role in the pathophysiology of obesity-associated hypertension. They suggested that both the RAAS and the natriuretic peptide system contribute to these alterations [34].

Sodium retention in obese hypertensive patients can be related to dysregulation of the RAAS which may be associated with the reduced level of cardiac natriuretic peptides. The abnormal RAAS regulation in obesity can result from a primary increased production of RAAS components and/or from a secondary increase due to a defective natriuretic peptides system [35].

In obese and nonobese hypertensive patients, the addition of NaCl was not associated with significant decrease in AngII or in aldosterone levels in spite of a significant increase in serum Na in obese patients and a highly significant reduction in serum K in nonobese patients. This may indicates that the addition of NaCl failed to suppress AngII and aldosterone in this group of nonobese hypertensive patients. In nonobese hypertensive patients, we found that serum Na had significant positive correlation with serum insulin after intake of glucose with NaCl. Oppermann et al. suggested that a constant exposure of the macula densa cells to salt concentrations may lead to some desensitization of the macula densa cells [36], but this remains to be further investigated.

It is well known that subjects of African origin are naturally predisposed to Na retention. Historically this was linked to survival advantage in extremes of Na scarcity; but in the modern environment where Na intake exceeds recommended intake, this leads to counter-regulation of the RAAS with suppression of renin and aldosterone and stimulation of ANP to be normotensive [37]. Subjects in this study are from East and West African origin. We hypothesize that failure of suppression of aldosterone and AngII by salt intake together with the antinatriuretic effect of insulin may provide a mechanism for development of essential hypertension. In this study, we did not assess salt sensitivity or measure the renin level; therefore, this hypothesis will need more focused investigation.

In this study, serum aldosterone was significantly higher in hypertensive patients than in control subjects. Comparable results were reported by Rossi et al. who found that plasma aldosterone was elevated in hypertensive obese subjects and it correlated positively with BMI in patients with essential hypertension but not in those with primary aldosteronism. They suggested a pathophysiological link between visceral adiposity and aldosterone secretion [38]. To verify the relation between obesity aldosterone and hypertension, we compared aldosterone level in untreated obese and nonobese hypertensive patients and their matched control subjects at baseline and after intake of the same amount of glucose alone and with NaCl. We found that obese hypertensive patients had higher serum aldosterone compared with their matched normotensive control subjects. The same finding was reported in nonobese group which showed higher aldosterone levels in nonobese hypertensive patients compared with their matched control subjects. This suggests that aldosterone may have a role in pathophysiology of essential hypertension, especially in obese patients.

In hypertensive patients, aldosterone levels were unexpectedly higher in nonobese than obese patients which were significant after intake of glucose alone. This could be attributed to the small sample size or to the intake of glucose. Intake of glucose increased insulin level which was higher in obese subjects. This may lead to entrance of K inside the cells, resulting in lower serum K level in obese compared with nonobese patients. The higher K level in nonobese patients may be the cause of higher aldosterone compared with nonobese patients.

In normotensive, serum aldosterone levels were significantly higher in obese normotensive subjects than nonobese subjects after intake of glucose and glucose with NaCl. Comparable results were reported by Bentley-Lewis et al. who found that aldosterone production is increased in normotensive overweight subjects compared with lean normotensive adults [39]. It was suggested that human adipocytes secrete potent mineralocorticoid-releasing factors, which suggest a direct link between obesity, insulin resistance, and hypertension [40]. Recently, mineralocorticoid receptor antagonists have shown considerable beneficial effects in hypertension [41], heart failure [42], metabolic syndrome [43], chronic kidney disease [44], atherosclerosis [45, 46], and vascular diseases [47]. This reflects the important role of aldosterone in the pathophysiology of hypertension, metabolic syndrome, and chronic kidney disease and raises the possibility of wider use of these drugs.

In this study aldosterone showed significant positive correlation with BP not only in obese hypertensive patients but also in nonobese normotensive subjects after intake of glucose alone. Vasan et al. reported that serum aldosterone level was related directly to BP outcomes in normotensive subjects after four years of follow-up. They suggested that high plasma aldosterone concentrations within the physiological range in nonhypertensive subjects increase the risk of subsequent development of hypertension [48]. Kaplan reported that many patients with hypertension have evidence of excess aldosterone production. Primary aldosteronism was present in 5–40% of all hypertensive patients [49]. Grim et al. showed that plasma aldosterone and the aldosterone/renin ratios were higher in the hypertensive compared to normotensive subjects [50]. They found that BP correlated positively with plasma aldosterone. However, correlations of BP with aldosterone were more consistent and more striking in subjects of African origin than in French Canadians. They suggested that aldosterone-induced volume expansion is an important contributor to hypertension, especially in blacks [50].

We suggest that aldosterone may have a role in short-term regulation of BP. The rapid nongenomic effects of aldosterone have been suggested to have a role in the control of vascular resistance and consequently in human hypertension and cardiovascular disease [14]. Rapid nongenomic effects of aldosterone have been reported in the heart [51], the colon [52], and the kidneys [53]. Furthermore, Wang et al. found that in the brain, mineralocorticoids activate brain sodium channels, with small increases in cerebrospinal fluid (CSF) Na^+^, leading to increases in brain ouabain-like compounds, sympathetic outflow, and BP [54]. Amin et al. showed that epithelial Na channels (ENaC) and mineralocorticoid receptors were present in the cardiovascular regulatory centers in the rat brain. They suggested that these channels may be regulated by aldosterone [55]. Leenen hypothesized that in salt-sensitive hypertension, an increase in CSF [Na^+^] causes a local increase in aldosterone biosynthesis which enhances the activity of angiotensinergic sympathoexcitatory pathways, leading to hypertension [56].

## 5. Conclusion

In obese hypertensive patients, the intake of glucose with NaCl was associated with a significant increase in serum Na and higher serum AngII compared with nonobese patients. These findings support the hypothesis that Na retention caused by increased AngII, probably from adipose tissue origin [25], may play a central role in the pathophysiology of obesity-associated hypertension.

In this group of hypertensive patients, oral intake of glucose and glucose with NaCl was not associated with significant decrease in AngII or in aldosterone levels in spite of a significant increase in serum Na in obese patients and a highly significant reduction in serum K in nonobese patients after intake of glucose with NaCl. This may indicates that dysregulation or failure of suppression AngII and aldosterone may play a role in the pathophysiology of essential hypertension.

## Figures and Tables

**Table 1:** Comparisons of the means of studied variables between obese and nonobese hypertensive and control subjects at baseline (fasting state).

Variables at fasting state	Nonobese patients mean ± S.E (*n* = 9)	Obese patients mean ± S.E (*n* = 11)	*t*-test *P* value	Nonobese control mean ± S.E (*n* = 7)	Obese control mean ± S.E (*n* = 8)	*t*-test *P* value
Systolic BP (mmHg)	150.7 ± 4.2	151.8 ± 4.4	0.85	119.1 ± 5.1	119.8 ± 2.2	0.92
Diastolic BP (mmHg)	98.1 ± 3.3	100.8 ± 5.6	0.68	80.0 ± 3.0	79.6 ± 1.6	0.92
Mean BP (mmHg)	115.7 ± 3.4	117.9 ± 5.1	0.72	93.0 ± 3.6	93.1 ± 1.8	0.98
Plasma glucose (mg/dL)	80.6 ± 3.2	82.0 ± 3.6	0.77	85.4 ± 5.9	93.0 ± 4.7	0.34
Serum Na^+^(mmol/L)	135.0 ± 1.8	138.2 ± .9	0.17	139.4 ± 1.6	141.0 ± 1.4	0.47
Serum K^+^ (mmol/L)	3.8 ± 0.1	3.9 ± 0.1	0.63	3.9 ± 0.1	4.1 ± .1	0.18
Serum insulin (*μ*IU/ml)	14.6 ± 2.8	15.5 ± 2.0	0.80	8.9 ± 0.7	9.4 ± 1.3	0.70
Serum aldosterone (pg/ml)	205.9 ± 20.1	191.7 ± 14.5	0.566	126.6 ± 12.6	167.6 ± 10.4	0.02^*∗*^
Serum angiotensin II (ng/ml)	31.7 ± 0.48	35.2 ± 2.1	0.153	—	—	—

**Table 2:** Serum angiotensin II, aldosterone, Na, K insulin, and plasma glucose in obese hypertensive patients after intake of glucose with NaCl.

Parameter	Means ± SE					
	Fasting	30 min	60 min	90 min	120 min	*P* value
Serum angiotensin II (ng/ml)	35.2 ± 2.1	34.5 ± 2.0	33.3 ± 1.9	33.7 ± 1.9	34.2 ± 1.8	0.968
Serum aldosterone (pg/ml)	191.7 ± 14.5	172.4 ± 17.7	168.4 ± 13.9	168.7 ± 14.2	146.5 ± 8.7	0.285
Serum Na (mmol/L)	138.2 ± 0.9	142.3 ± .6	140.0 ± 1.1	138.82 ± 1.0	138.36 ± 1.1	0.034
Serum K (mmol/L)	3.9 ± 0.1	3.6 ± .1	3.5 ± 0.1	3.6 ± 0.1	3.6 ± 0.1	0.134
Plasma glucose (mg/dL)	84.9 ± 5.2	141.5 ± 9.7	136.1 ± 8.4	125.8 ± 9.6	103.5 ± 7.5	0.000
Serum insulin (*μ*IU/ml)	16.9 ± 3.2	61.0 ± 7.5	77.0 ± 12.4	66.0 ± 15.0	39.8 ± 8.1	0.001

**Table 3:** Serum angiotensin II, aldosterone, serum Na, K insulin, and plasma glucose in nonobese hypertensive patients after intake of glucose with NaCl.

Parameter	Means ± SE					
	Fasting	30 min	60 min	90 min	120 min	*P* value
Serum angiotensin II (ng/ml)	31.7 ± 0.4	33.8 ± 2.7	30.7 ± 1.0	30.6 ± 1.1	30.8 ± 0.7	0.522
Serum aldosterone (pg/ml)	205.9 ± 20.1	191.1 ± 18.1	185.9 ± 17.4	167.5 ± 12.3	171.6 ± 16.5	0.488
Serum Na (mmol/L)	135.0 ± 1.8	138.7 ± 1.3	138.0 ± 1.0	137.8 ± 0.8	138.1 ± 1.3	0.313
Serum K (mmol/L)	3.8 ± 0.15	3.3 ± 0.09	3.3 ± 0.07	3.3 ± 0.07	3.4 ± 0.06	0.001
Plasma glucose (mg/dL)	78.0 ± 4.4	133.3 ± 9.2	148.0 ± 13.0	129.6 ± 9.9	117.7 ± 11.7	0.000
Serum insulin (*μ*IU/ml)	12.7 ± 2.1	54.0 ± 8.88	60.3 ± 6.3	56.5 ± 4.4	45.6 ± 5.1	0.000

**Table 4:** Comparisons of the means of the studied parameters in hypertensive and normotensive control subjects after intake of glucose with NaCl.

Variable	Nonobese hypertensive mean ± S.E (*n* = 45)	Obese hypertensive mean ± S.E (*n* = 55)	*t*-test *P* value	Nonobese normotensive mean ± S.E (*n* = 35)	Obese normotensive mean ± S.E (*n* = 40)	*t*-test *P* value
Systolic BP (mmHg)	146.4 ± 1.0	146.3 ± 1.6	0.924	116.8 ± 1.9	123.3 ± 1.6	0.010^*∗*^
Diastolic BP (mmHg)	93.7 ± 1.4	93.5 ± 1.8	0.943	77.3 ± 0.90	80.2 ± 0.96	0.031^*∗*^
Mean BP (mmHg)	111.3 ± 1.3	111.1 ± 1.7	0.924	90.5 ± 1.2	94.6 ± 1.1	0.014^*∗*^
Serum Na (mmol/L)	137.56 ± 0.59	139.58 ± 0.48	0.009^*∗*^	139.7 ± 0.43	139.4 ± 0.56	0.688
Serum K (mmol/L)	4.1 ± 0.72	3.6 ± 0.05	0.48	3.8 ± 0.08	3.8 ± 0.05	0.024^*∗*^
Serum aldosterone (pg/ml)	185.98 ± 8.13	169.60 ± 6.38	0.111	109.25 ± 5.45	144.80 ± 5.2	0.000^*∗*^
Serum angiotensin II (ng/ml)	31.56 ± 0.65	34.29 ± 0.85	0.016^*∗*^	—	—	—

†Comparisons based on the mean value of 5 samples taken 0–120 minutes after intake of glucose with NaCl.

**Table 5:** Comparisons of the means of the studied parameters in hypertensive and normotensive control subjects after intake of glucose.

Variable	Nonobese patients mean ± S.E (*n* = 9)	Obese patients mean ± S.E (*n* = 11)	*t*-test *P* value	Nonobese control mean ± S.E (*n* = 7)	Obese control mean ± S.E (*n* = 8)	*t*-test *P* value
Systolic BP (mmHg)	148.2 ± 1.5	148.5 ± 2.1	0.93	119.1 ± 2.0	118.6 ± 1.6	0.82
Diastolic BP (mmHg)	93.6 ± 1.7	95.8 ± 2.3	0.46	78.2 ± 1.2	76.9 ± 1.1	0.43
Mean BP (mmHg)	111.9 ± 1.5	113.4 ± 2.2	0.60	91.8 ± 1.4	91.5 ± 1.1	0.58
Serum Na (mmol/L)	137.9 ± 0.58	138.5 ± 0.56	0.47	139.6 ± 0.5	140.7 ± 0.5	0.13
Serum K (mmol/L)	3.6 ± 0.1	3.7 ± 0.1	0.80	3.7 ± 0.1	3.9 ± 0.1	0.01^*∗*^
Serum aldosterone (pg/ml)	245.90 ± 16.1	200.55 ± 9.3	0.013^*∗*^	106.9 ± 8.5	171.6 ± 9.5	0.000^*∗*^
Serum angiotensin II (ng/ml)	28.10 ± 1.2	31.13 ± 1.52	0.136	—	—	—

†Comparisons based on the mean value of 5 samples taken 0–120 minutes after intake of glucose.

## Data Availability

The data used to support the findings of this study are available from the corresponding author upon request.

## Additional Points

In this study, we did not measure plasma renin or 24-hour sodium and potassium excretion. Small sample size could be one of the limitations of this study.
